# Chemically Modified Polyvinyl Chloride for Removal of Thionine Dye (Lauth’s Violet)

**DOI:** 10.3390/ma10111298

**Published:** 2017-11-12

**Authors:** Helena Ma A. M. M. S. Ali, Cleuzilene V. Silva, Betina Royer, Guimes Rodrigues Filho, Daniel A. Cerqueira, Rosana M. N. Assunção

**Affiliations:** 1 Institute of Chemistry, Federal University of Uberlândia, IQUFU, Av. João Naves de Ávila, 2121, Santa Mônica 38400-902, Uberlândia, MG, Brazil; helenaali@iftm.edu.br (H.M.A.M.M.S.A.); cleuzilene@gmail.com (C.V.S.); betiroyer@yahoo.com.br (B.R.); guimes.rodriguesfilho@gmail.com (G.R.F.); 2 Chemistry Department, Federal Institute of Education, Science and Technology of the Triângulo Mineiro, IFTM, Rua João Batista Ribeiro, 4000, D.I. II, Uberaba 38064-790, MG, Brazil; 3 Institute of Exact, Natural and Educational Sciences, ICENE, Federal University of Triângulo Mineiro, UFTM-Unit 2, Av. Randolfo Borges Júnior, 1250, Univerdecidade, Uberaba 38064-200, MG, Brazil; d.a.cerqueira@gmail.com; 4 Faculty of Integrated Sciences of Pontal, FACIP of Federal University of Uberlândia, UFU, R. Vinte, 1600, Tupã Ituiutaba 38304-402, MG, Brazil

**Keywords:** poli(vinyl chloride), sulfonation, adsorption, dye, environment

## Abstract

The chemical modification of hydrophobic polymer matrices is an alternative way to elchange their surface properties. The introduction of sulfonic groups in the polymer changes the surface properties such as adhesion, wettability, catalytic ability, and adsorption capacity. This work describes the production and application of chemically modified polyvinyl chloride (PVC) as adsorbent for dyes removal. Chemical modification of PVC was evaluated by infrared spectroscopy and elemental analysis, which indicated the presence of sulfonic groups on PVC. The chemically modified PVC (PVCDS) showed an ion exchange capacity of 1.03 mmol^−1^, and efficiently removed the thionine dye (Lauth’s violet) from aqueous solutions, reaching equilibrium in 30 min. The adsorption kinetics was better adjusted for a pseudo second order model. This result indicates that the adsorption of thionine onto PVCDS occurs by chemisorption. Among the models for the state of equilibrium, SIPS and Langmuir exhibited the best fit to the experimental results and PVCDS showed high adsorption capacities (370 mg^−1^). Thus, it is assumed that the system presents homogeneous characteristics to the distribution of active sites. The modification promoted the formation of surface characteristics favorable to the dye adsorption by the polymer.

## 1. Introduction

The polyvinyl chloride (PVC) is a versatile thermoplastic wide used in the world with a global demand exceeding 35 million tons per year [1]. Brazil is responsible for the consumption of around 2% of the global demand for PVC resins. About 66.0% of all PVC is utilized in tubes, connections, threads, and cables. In such applications, PVC is more economical than other materials, such as wood, metals, and ceramics. Moreover, it has advantages in terms of some fundamental requirements, such as anti-flame behavior, weathering resistance, thermal and acoustic isolation, ease of installation, low maintenance, and excellent finishing and aesthetic qualities. Despite the advantages of using PVC in building and construction, the high chlorine content in the polymer is a disadvantage from an environmental point of view, since 80% of organically bound chlorine and around 60% of total chlorine in municipal waste is associated with this polymer. Undesirable processes may occur with chlorinated polymeric materials like PVC and DPVC. PVC is a polymer with low thermal and photochemical stability. Under the action of the medium, especially at high temperatures, the PVC is degraded by dehydrochlorination of the polymer chains leading to the release of hydrochloric acid (HCl), with the formation of a polyene structure. The release of HCl is one of the major problems associated with PVC recycling since the generated vapors are toxic and lead to corrosion of treatment systems. In pyrolysis of PVC, for example, in addition to the generation of HCl, other chlorinated organic molecules can be produced, which make the products undesirable for use as feedstock or fuel [2,3]. The dehydrochlorination begins at thermally labile defect site in the polymer chain. When the concentrations of HCl and polyenes have reached a certain level, these products react to form polyenyl cation radicals that lead to autocatalysis [4,5]. The process leads to the discoloration of the polymer and in the presence of oxygen, oxidative reactions occur that lead to the cleavage of the macromolecular chains affecting the mechanical properties of the PVC due to the weakening of the material. These numbers show the necessity to reuse these polymeric wastes. When considering a minimization hierarchy, disposal in dumps should be the last alternative [1]. An interesting possibility as plastic waste recycling is an activation of PVC scrapt after removal HCl, and this result an active removal that is a good absorbent for methylene blue [6].

PVC can be modified by nucleophilic substitution of chlorine atoms in its structure and by elimination reactions, for dehydrochlorination, and for the formation of conjugated double bonds [7]. This transformation is interesting as it can extend the possibility of PVC reuse and decrease the toxicity associated with chlorine. PVC dehydrochlorination products (DPVC) can be obtained through polymer thermal decomposition, photochemistry reactions by ionizing radiation, or basic catalysis. These modifications result in the elimination of hydrogen chloride and cause changes in the polymer color, which turns orange-brown according to the dehydrochlorination degree.

Our research group [8] adapted the process utilized by Guo et al. [9] for PVC dehydrochlorination to produce chemically-modified PVC by dehydrochlorination reactions followed by treatment with sulfuric acid. The modified PVC was obtained as a brown powder, partially dehydrochlorinated, with the insertion of polar groups (carbonyl and hydroxyl), and possibly sulfonic acid groups. The ion exchange capacity of the chemically modified material reached 1.30 mmol·g^−1^ of dry polymer. This method can be used to modify the PVC film surface leading to the presence of hydroxyl and carbonyl terminal groups and sulfonic groups. The presence of these groups allows for the immobilization of biomolecules such as heparin, insulin, amino acids, and enzymes, and the adsorption of active substances such as drugs, fertilizers, and herbicides. Xu and Lee (2009), have sulfonated PVC to produce PVCSO_3_H and employ it directly as an individual device for extraction purposes in the cation-exchange microextraction of anaesthetics. The sulfonated PVC showed a sulfur content of 2.66% and the ion exchange capacity was estimated as 1.71 mmol·g^−1^ [10]. Change in the characteristics of the polymer matrix can be achieved by chemical surface modification or modification of bulk polymer.

Chemically modified polymers can be employed as adsorbent due to the presence of sulfonic and hydroxyl groups and the hydrophobic polymer chain creates a surface-active material with potential for use as ion exchanger, catalyst in esterification reactions, and adsorbent for complex organic molecules like dyes [1,7,8,9,11]. The chemical modification of the adsorbent plays an important role in the increase in the adsorption of dyes. Gupta et al. (2012) developed from ground tire granules that were activated to 900 °C, a chemically active material by treating the adsorbent with nitric acid. This material has functional groups with oxygen, where the fixation of these groups leads to the preparation of a material with hydrophilic active sites [12]. The adsorbent presented superior adsorption capacity to the original material without treatment with nitric acid. The presence of chemical groups of activated carbon surface substantially increases the dye removal capacity [13]. According to Nayak et al. (2017), chemical activation is known to induce specific surface features of porosity and functionality, which play a definite role in enhancing the adsorptive potential of the developed activated carbons [14]. The modified PVC has a chemically active surface that enables adsorption of polar molecules, such as dyes. The characteristics are very close to those observed for the mentioned systems, in this sense, we believe that this polymer will present excellent performance in adsorption processes of polar molecules as dyes.

Dyes have been used for thousands of years as textile paints and pigments. There is currently a huge variety of paints and pigments commercially available. The production of dyes reaches values in the order of 1.6 million ton and 10 to 15% of this volume is discarded. In this sense, dyes are water pollutants. Dyes are complex organic molecules that are non-degradable that have good chemical stability and are difficult to remove. These molecules can be classified in relation to their chemical structure (azo, anthraquinone, Indigoid, nitro, triarylmethane, and thiamine dyes), color and applications methods. In relation to the chemical structure, dyes are frequently classified in relation to the charge they present in aqueous solution, such as cationic, anionic, and nonionic dyes [15].

Wastewater treatment in the textile industry aims to reduce the color and presence of dissolved organic molecules. Various methods can be employed for the treatment of effluents such as: chemical precipitation, ion exchange, coagulation/flocculation, flotation, membrane filtration, electrochemical treatment, and adsorption. Several of the cited methods have advantages and disadvantages that must be considered in terms of cost and performance. Among them, adsorption is a process where the accumulation of adsorbate occurs on the porous surface of the adsorbent. This method presents as advantages a high efficiency of removal of pollutants at very low concentrations. The adsorption process is the most popular because they are cost-effective and present adequate efficiency, being the performance dependent on the choice of the adsorbent [15,16,17].

In this context, this work demonstrates the application of chemically modified polyvinyl chloride as potential adsorbent for the removal of the dye thionine (Lauth’s violet) from aqueous solutions. The chemical modification of PVC was investigated by infrared spectroscopy and elemental analysis and the measurement of the ion exchange capacity was determined. The adsorbent was characterized by thermogravimetry, X-ray diffraction, surface area measurements and scanning electron microscopy. Kinetics and adsorption isotherms were studied to characterize the dye adsorption process on the new adsorbent.

## 2. Results and Discussion

### 2.1. Adsorbent Characterization

**Figure 1:**
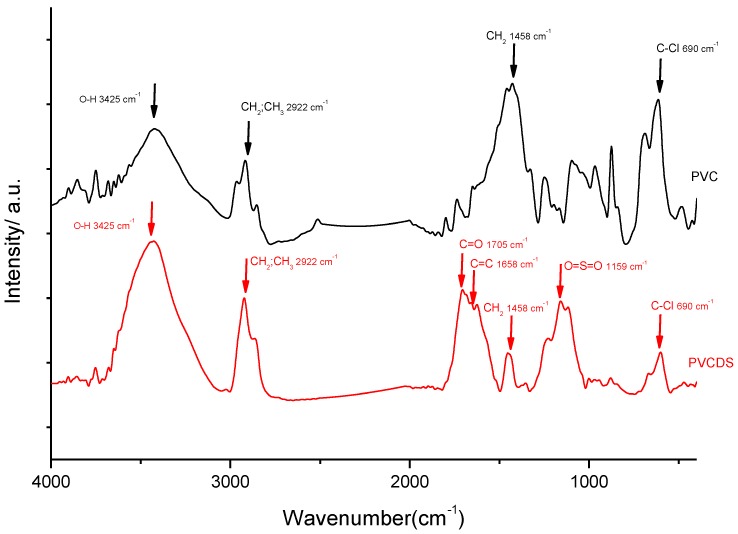
Infrared absorption spectroscopy for the unmodified polyvinyl chloride (PVC) and after the chemical modification (PVCDS).

The absorption spectra in the infrared region, shown in Figure 1 (PVC and PVCDS curves) for PVC reveals the presence of at least three peaks in the region between 3000 and 2800 cm^−1^, with the most intense being centered at 2922 cm^−1^, attributed to the axial deformation of the C-H bond, symmetrical and asymmetric modes, present throughout the carbon chain. The chemical modification of the PVC leads to change in the profile of this band due to the elimination reactions since it may be related to the output of chlorine atoms and a proportional increase in quantitative carbon (sp^2^) in the structure. The presence of chlorine in the polymer structure before and after the chemical modification is confirmed by the presence of a peak at 690 cm^−1^ attributed to the axial deformation of the C-Cl bond. When comparing the profiles of the PVC and PVCDS infrared curves, the decrease of chlorine in the PVDCS structure is clearly observed, confirmed by the reduction in the intensity of this band.

**Scheme 1:**
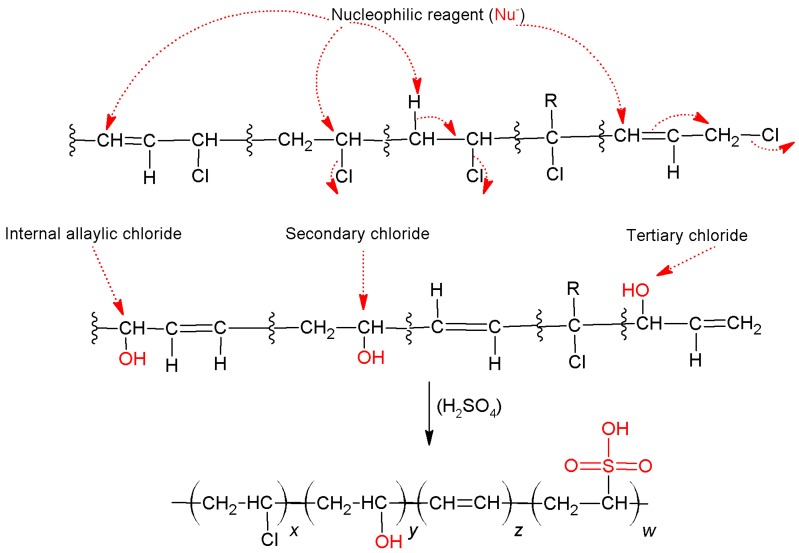
Mechanism for the dechlorination and sulfuric acid treatment of PVC.

It is also observed, for both samples, the presence of the peak at 3425 cm^−1^, assigned to the presence of O-H hydroxyl groups, in the structure. Although unexpected, the presence of this band for PVC may be related to additives added during the synthesis of the polymer [18]. For the PVCDS, the increase in the intensity of this band is related to the chemical modifications undergone during the process of dehydrochlorination and subsequent treatment with sulfuric acid. Dehydrochlorination leads to electrophilic elimination, with the formation of polyenic structure [8]. This process is followed through competitiveness of a nucleophilic substitution, which leads to the formation of hydroxyl groups. The spectrum in the infrared region for the PVDCS shows the presence of a peak at 1705 cm^−1^, attributed to the formation of carbonyls that may be associated with oxidative processes and a peak at 1658 cm^−1^, assigned to C=C stretching indicating elimination reactions during treatment with concentrated sulfuric acid. The treatment with sulfuric acid leads to the appearance of some intense bands in the region between 1200 and 1100 cm^−1^, particularly the band at 1159 cm^−1^, assigned to stretching of O=S=O resulting from the formation of sulfonic acid group in the polymer [19], as shown in Scheme 1 [20]. The PVCDS has similar structures to others sulfonated polymeric materials.

The chemical modification of PVC was also confirmed by the elemental analysis data presented in Table 1. The percentage of chlorine in the PVC structure was evaluated through the difference of the values found for carbon and hydrogen, and the value found was 59.68% of mass. This value is higher than expected when considering that the percentage by mass of chlorine in the monomeric unit of PVC is equal to 56.73%. The difference observed, 2.95%, may be related to additives in their structure.

The percentage of sulfur atoms in the chemically modified sample (PVDCS) significantly increased in polymer structure and these results are confirmed by FTIR data. 

The conversion of the chemical modification reaction of PVC was evaluated through the reduction of the chlorine amount in the polymer found from the *Cl*/*C* molar ratio obtained for the PVC, considering the monomer unit of the polymer as compared to the values of *Cl*/*C* molar ratio for PVCDS, according to Equation (1), proposed by Guo et al. [9].

(1) $$\left(DCI\right)=\;2\left[0.50\;-\;{\left(\frac{Cl}{C}\right)}_{p}\right]\times \;100$$

where (*Cl*/*C*)*p* is the ratio obtained from chemically modified samples.

When considering the data presented in Table 1, the reaction presents 65% of dehydrochlorination. It is observed from the elemental analysis data the presence of sulfur atoms in the structure, at a ratio of about 31 carbon atoms for each sulfur atom, or considering the monomeric unit of PVC, wherein for every 16 units, there is one unit with a sulfur atom attached.

To evaluate the chemical properties of the groups bound to the modified PVC, the ion exchange capacity was investigated. According to Siva, et al. (2012), the ion exchange capacity for pure PVC is of the order 0.044 mmol·g^−1^ (±0.02) that was already performed for the chemically modified PVC, at a temperature of 300 K (±1) and pH 5.0 (±0.5) resulted in an ion exchange capacity of 1.03 mmol·g^−1^ (±0.03) [21]. The assay performed at a temperature of 300 K (±1) and pH 5.0 (±0.5) resulted in an ion exchange capacity of 1.03 mmol·g^−1^ (±0.03). This result indicates the possible presence of sulfonic groups responsible for the ion exchange process. The obtained result is like previous work developed with similar materials [8].

The values of surface area, *σ*, specific volume of pores, *V*, and average diameter of pores, *d*, of original PVC and PVCDS were obtained through adsorption and desorption measurements of N_2_ at −196.6 °C. The results obtained are presented in Table 2.

The surface area, total pore volume, and average pore diameter of the PVCDS were 295.57 m^2^·g^−1^, 0.43 cm^3^·g^−1^, and 5.76 nm, respectively. The average pore diameter of 5.76 nm indicated that the PVCDS was in the mesoporous region. The increase of surface area and pore volume of the PVCDS possibly occurs due to treatment with sulfuric acid leading to a change in surface roughness as observed by Xu and Lee (2009) in the SEM micrograph [10]. 

**Figure 2:**
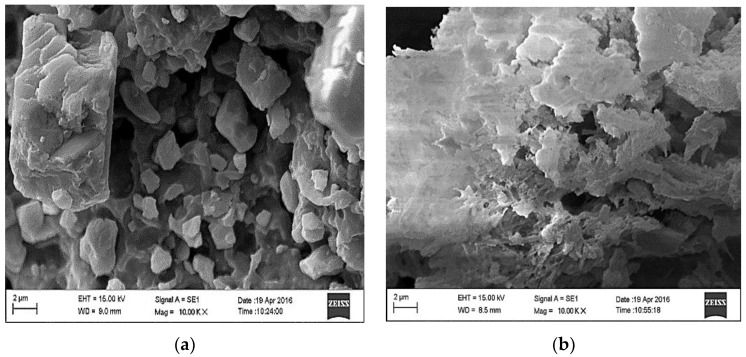
Scanning electron microscopy for: (**a**) polyvinyl chloride without modification (PVC) and (**b**) after chemical modification (PVCDS).

Figure 2a,b show the morphology of the PVC and PVCDS, respectively. As it can be seen from Figure 2a, the original PVC has granular structure, with microparticles of different sizes and dense appearance. After the chemical modification, the morphological structure of PVC changes significantly with the formation of possible lamellar regions, apparently represented by small scale flakes. Regions that distinguish among scales are observed with possible porous structure, Figure 2b. The chemical treatment of PVC modifies its original morphology and leads to an increase of the surface area, desired aspect for the use of this material as an adsorbent.

**Figure 3:**
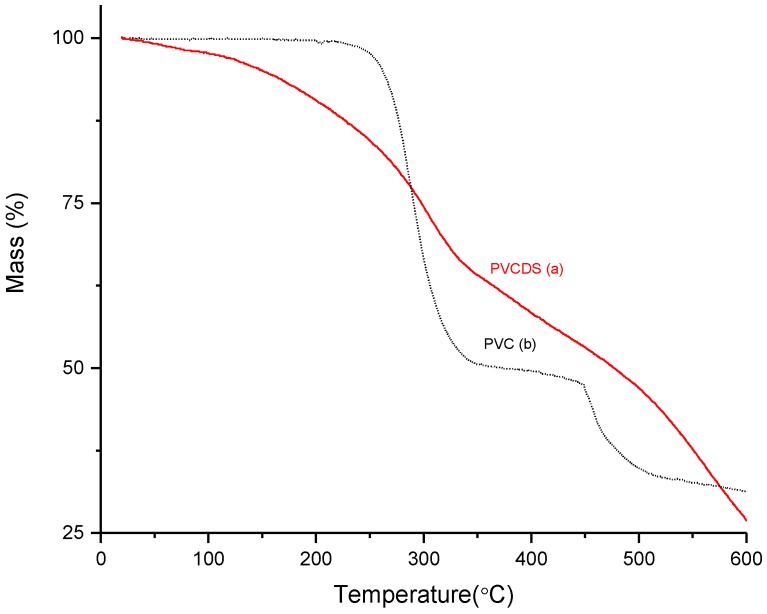
Thermogravimetric curves: (**a**) polyvinyl chloride without modification (PVC) and (**b**) after chemical modification (PVCDS).

The thermal stability of the modified PVC was evaluated through thermogravimetric curves, as shown at Figure 3. The original PVC presents three thermal events: (i) between 240 and 350 °C, attributed to thermal dehydrochlorination, with a reduction of about 56% in mass. The percentage of chlorine reduction corroborates the data for elemental analysis of the material; (ii) the second event started at approximately 450 °C is related to chain rupture after the condensation process and chain rearrangement reactions, and (iii) a third event can be observed and is assigned to decomposition of carbonaceous residues observed between 550 °C and 600 °C.

For the PVDCS, when considering that most chlorine present in the structure was removed by chemical dehydrochlorination, the thermal events are related to the removal of remaining chlorine (thermal dehydrochlorination) and other processes such as desulfonation, which leads to a decrease in the thermal stability of the modified polymer since materials with sulphonic groups exhibit decomposition between 200 °C and 400 °C [22,23,24]. The obtained curves resemble those observed for other sulfonated materials. Other thermal events are observed at temperatures above 400 °C and may be related to the decomposition of the polymer due to the cleavage of the main chain.

**Figure 4:**
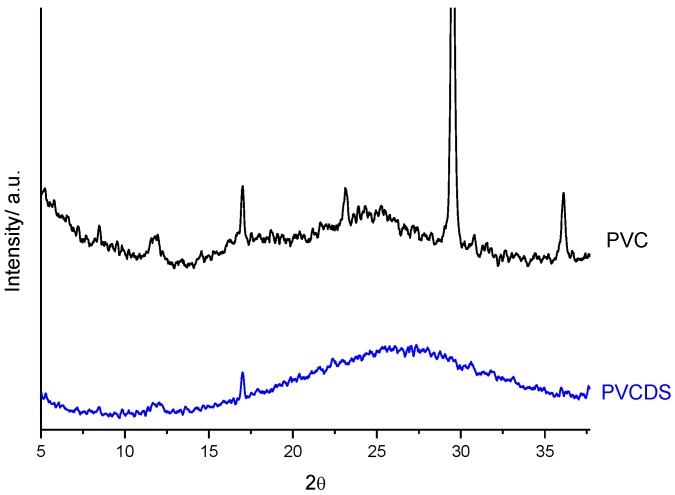
X-ray diffraction patterns: Polyvinyl chloride (PVC) and PVC after chemical modification (PVCDS).

Figure 4 shows the X-ray diffraction for PVC and PVCDS. For PVC, it was observed the presence of the one peak at 2θ equal to 16.9° and halo centered at 24.6° 2θ, the first one associated to the crystalline regions of the PVC and the last one evidences the amorphous character of the original material [25]. The polymer is semicrystalline, presumably showing to have a low crystallinity index which can be identified by the diffractogram profile.

The chemical modification of the PVC changes the diffraction pattern of the derivative produced (PVCDS), increasing the area of the halo between 19° and 25°, 2θ. This aspect indicates an increase in the amorphous fraction of the polymer. It is also observed a reduction in peak intensity at 17°, 2θ, which corroborates the previous assertion. The reduction of crystallinity increases the accessibility of the polymer, which can improve the adsorption process.

### 2.2. Adsorption Kinetics

#### 2.2.1. Effects of pH on Adsorption

The pH of the solution is a significant parameter that affects the adsorption process of the dye. The acquaintance of the superficial charge of the adsorbent with the dye molecule is important tools for the knowledge of the adsorption mechanism [26]. Adsorption tests as function of pH were carried out with 25 cm^3^ of 20.7 mg·dm^−3^ VL aqueous solution, 10 mg of adsorbent and initial pH varied from 2 to 10.

**Figure 5:**
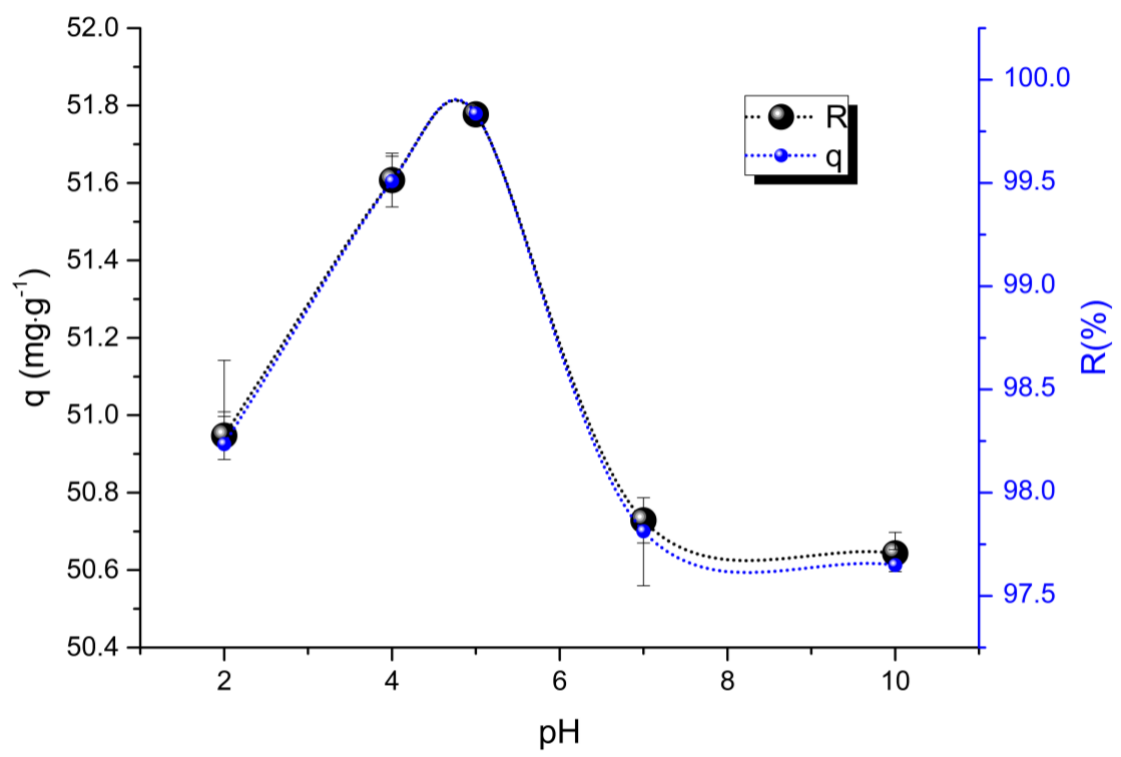
Influence of pH on adsorption capacity and removal rates.

Figure 5 shows the effect of pH on dyes adsorption.

The presence of the sulfonic groups, among others as carbonyls, hydroxyls, on the surface PVCDS, contributes to the reaction with the basic dye. The removal of VL dye throughout the studied pH range is higher than 97.0%. However, slight influence of pH is observed, since the maximum removal (~100%) of the dye occurs at pH equal to five. 

Removal of the VL increases with an increasing pH, reaches a maximum point at pH equal to 5, and begins to decrease to higher pH values. In acid medium, the surface of the adsorbent is protonated and in addition, the H^+^ ions compete for the active sites on adsorbent, leading to decrease of Dye removal, with the decrease of the concentration of these ions the adsorption of the dye increases. At pH 5, the adsorbent is deprotonated, and thus electrostatic forces between the negative net charge surface and the cation dye are established leading to 100% of dye removal. The increase in pH keeps the deprotonated adsorbent surface and the dye is protonated, this condition again decreases the interaction between both with reduction in the percentage of removal.

Therefore, the pH ≈ 5.0, is most favorable for the removal of basic dyes such as Lauth Violet, for the system used. In this pH range, there is an increase on the active sites in the resin, for the adsorption of the VL. As is well known, in adsorption processes, its efficiency depends both on the available active sites and on the adsorbent and adsorbent properties [27,28].

#### 2.2.2. Adsorbent Mass

**Figure 6:**
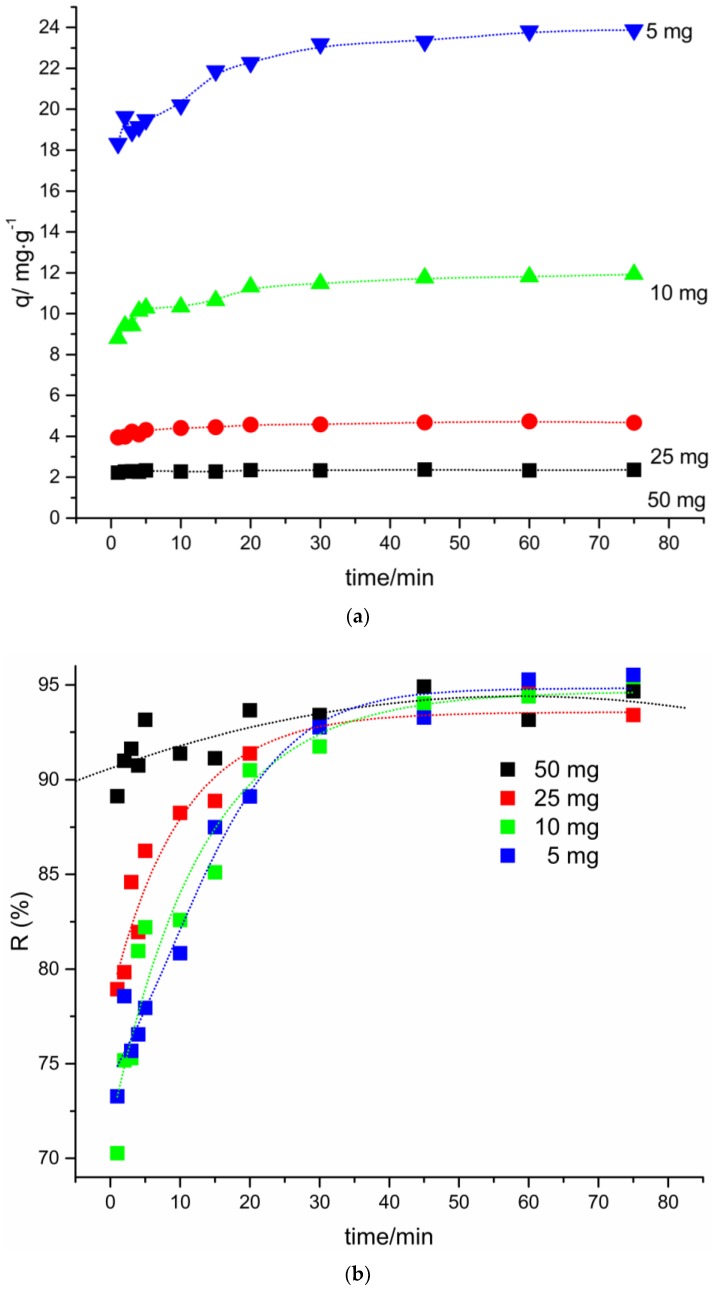
Effect of adsorbent dosage on (**a**) adsorption capacity and (**b**) percentage of removal.

Figure 6 shows the adsorbent (PVCDS) dosage effect on adsorption of VL. The study was carried out using adsorbent masses between 5 and 50 mg, dye solution concentration 5.0 mg·dm^−3^, and volume of 25 cm^3^.

In Figure 6a, the effect of the adsorbent mass on the change in adsorption capacity, *q*, was observed. The increase of adsorbent mass leads to a decrease in the adsorption capacity. The adsorption capacity considers the unit mass in grams of adsorbent, in this sense, for high adsorbent mass, several active sites remain unoccupied during the adsorption process. This result can also be explained by the overlap of the active sites due to the increase in the number of particles with increase of mass of the adsorbent [29].

The percentage of removal, *R* (%), Figure 6b, however, increases when the adsorbent mass is increased, reaching high values for the range studied. In this case, as there are active sites available, the dye molecules present in the solution are adsorbed at these sites, increasing the dye removal with increasing adsorbent mass.

One of the purposes of the study of the effect of adsorbent dosage is to obtain information on the effectiveness of the adsorption process and the ability of the dye to be adsorbed with the minimum dosage of the adsorbent. 

**Figure 7:**
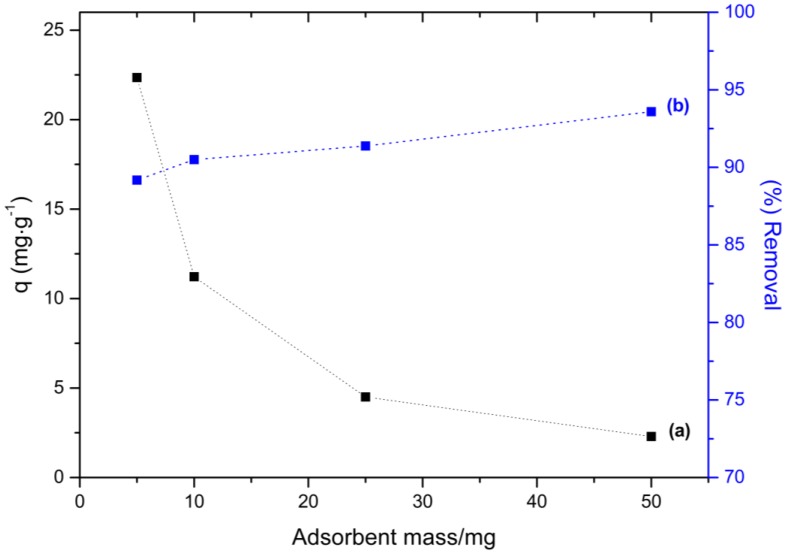
Effect of the dosage of adsorbent on (**a**) the adsorption capacity, *q* and (**b**) percentage dye removal.

When considering the dye initial concentration, the mass of 50 mg of adsorbent was high, leading to high percentage of dye removal, however with a low adsorption capacity, since there are several unoccupied sites. For lower adsorbent masses, the percent removal values are still high and an increase in adsorption capacity is observed. In the profile observed in Figure 7, the adsorbent mass used in the subsequent kinetics tests were 10 mg, since in this condition a percentage of removal of more than 90% and an adsorption capacity of 11.15 mg·g^−1^ were observed. It is important to mention that, in this condition, possible overlapping of the active sites is avoided due to aggregation phenomena occurring at high adsorbent concentrations. 

#### 2.2.3. Kinetics Models

Adsorption kinetics studies are important because they provide information about mechanism of adsorption process. 

**Figure 8:**
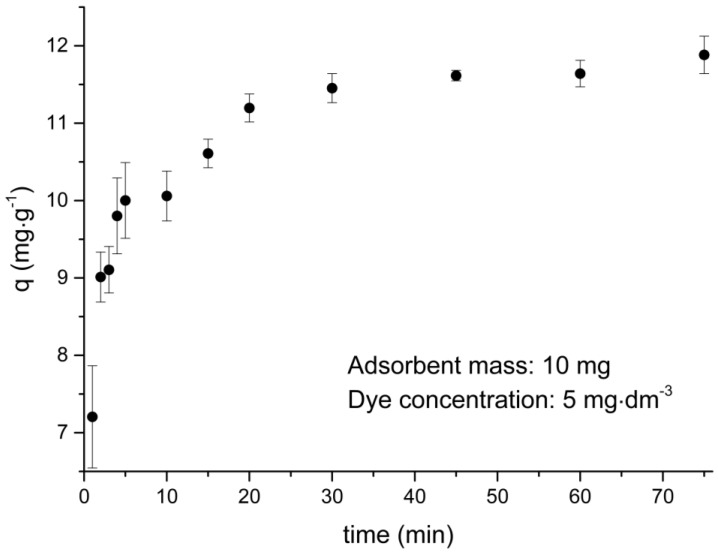
Adsorption kinetics of thionine dye (VL) dye.

The adsorption process was fast in the first 10 min, as can be seen in Figure 8. After this period, a decrease in the adsorption rate is observed until equilibrium is reached. This profile is expected since at the beginning of the process there are many active sites available for adsorption. This number of active sites decreases as the dye adsorption progresses, becoming difficult to occupy due to the increase of repulsive intermolecular forces between the dye molecules on the surface of the adsorbent.

The pseudo first-order, pseudo second-order, and intraparticle diffusion models were adopted to fit the experimental data and elucidate the kinetic adsorption mechanism. The equations and kinetic parameters pertinent to each model are shown in Table 3. 

**Figure 9:**
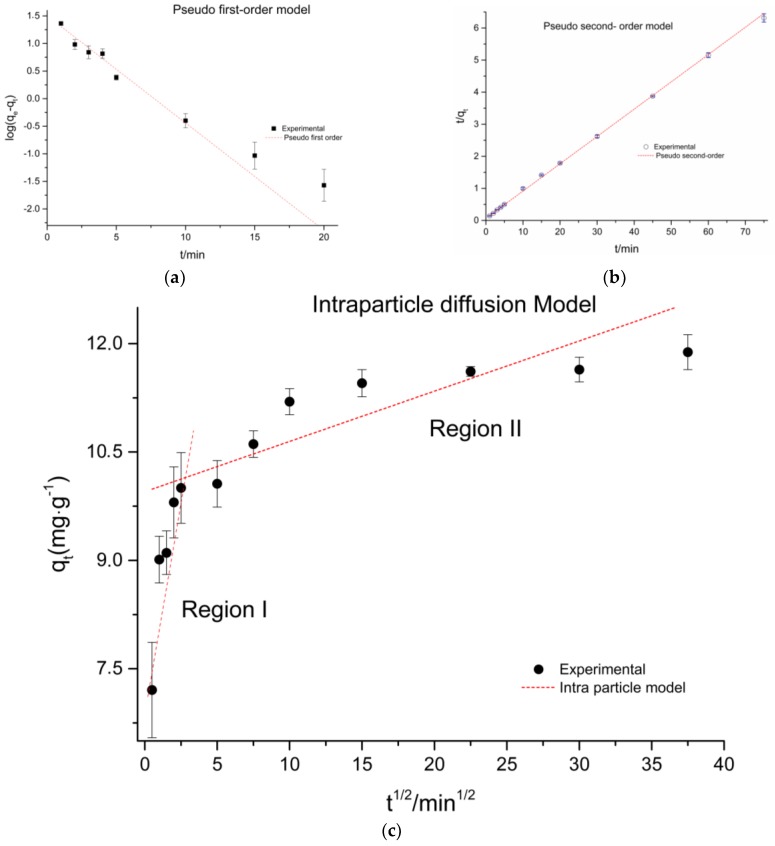
Kinetic models: (**a**) Pseudo-first order model; (**b**) Pseudo-second order model; (**c**) Intraparticle diffusion model.

Figure 9 presents the experimental data and the mathematical adjustment of the models that were adopted in this work. Table 4 summarizes the results obtained from the adjustment of the applied kinetic models.

Figure 9a shows the application of pseudo first-order model to VL adsorption on PVCDS. Value of *k*_1_ was obtained through slope of linear plot of log(*q_e_*−*q_t_*) versus *t* (shown in Table 4). The values of the determination coefficient, *R*^2^ and the adsorption capacity at equilibrium, *q_e_*, were, respectively, 0.94643 and 31.88 mg·g^−1^. The value of the experimental adsorption capacity was 11.955 mg·g^−1^, this value is lower than that theoretically obtained in the fit for the pseudo first order model.

The pseudo second order model was applied to the experimental data, as shown in Figure 9b. A better adjustment of the theoretical curve visually to the experimental data is observed. This observation is confirmed by the value of *R*^2^ that was 0.99936. In addition, the adsorption capacity at equilibrium was 11.74 mg·g^−1^, like the experimental data 11.95 mg·g^−1^. As it can be seen in Table 4, the pseudo-second order kinetic model was the one that better fits the experimental data, presenting the value of the determination coefficient close to 1 and the value obtained for the adsorption capacity at equilibrium very close to the experimental value. It is important to note that the pseudo second order model describes the chemosorption mechanism. In the adsorption process, the rate of reaction is dependent on the amount of solute adsorbed on the surface of the adsorbent and on the amount adsorbed at equilibrium. In this model, the rate-limiting step is due to the chemical adsorption that may involve valence forces by sharing or exchanging electrons between the dye and the adsorbent. In this case, it involves valency forces through the sharing or exchange of electrons between adsorbent and adsorbate, covalent forces, ion exchange, and electrostatic forces [30]. 

The dye molecules are possibly carried from the solution to the solid adsorbent through the intraparticle diffusion process. In Figure 9c, the intraparticle diffusion model was used to identity a possible mechanism of intraparticle diffusion as a limit step. 

The results obtained for the intraparticle diffusion model can be observed in Figure 9c and in Table 5. The curve can be interpreted considering several kinetic steps where a linear region is observed for each one. This result implies that the adsorption process for the VL involves more than one kinetic stage or adsorption rate [31]. The adsorption exhibited two stages, which can be attributed to two linear parts, as shown in Figure 9c.

In region (I), the adsorption capacity reaches 10.04 mg·g^−1^ in 5 min of assay. This indicates that adsorption occurs rapidly in the first stage due to the existence of unoccupied active sites and the occupation of mesopores. In the region (II), the adsorption rate decreases significantly, possibly due to the decrease of the active sites and the accommodation of the molecules in microporous regions. For the system under study, PVCDS, the first stage is predominant, since most of the dye is adsorbed in the first 10 min, reaching almost the adsorption capacity at equilibrium.

### 2.3. Adsorption Isotherm

Adsorption isotherms describe the relationship between the amount of substance that is adsorbed by an adsorbent and the concentration of the solution substance in equilibrium. The equilibrium studies provide, through models, an idea of the adsorption mechanism. The equations and isotherm parameters pertinent to each model are shown in Table 5. The application of these models to the experimental data is presented in Figure 9 and the parameters obtained are presented in Table 6. 

The evaluation of the parameters of the expressions representing each model (expressions of 2 or 3 parameters) provides a series of physico-chemical information such as adsorption capacity, surface properties, adsorbent affinity, and the interactions between adsorbate and adsorbent can be better understood [11]. 

**Figure 10:**
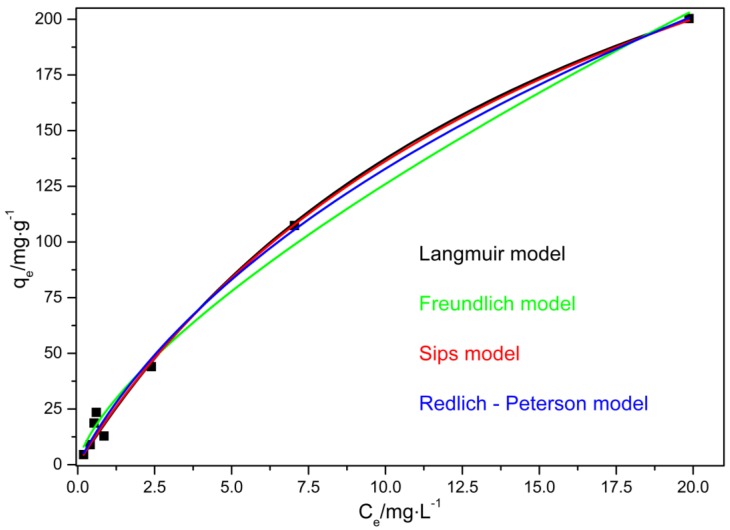
Application of the adsorption isotherm models to the experimental data.

Figure 10 shows the application of the adsorption isotherm models to the experimental data.

The Langmuir and Freundlich isotherm are two-parameter models that provide information about adsorption capacities and activation energies of the adsorption process. The isotherms models that consider these three parameters are obtained from the articulation of the expressions of the Langmuir and Freundlich models, such as the Redlich-Peterson and Sips models.

As seen in Figure 10, adsorption increases with an increasing concentration of VL at low dye concentrations, and tends to a near constant value at high dye concentrations. The results show that for the experimental conditions evaluated all the models present good adjustment of the experimental data since the coefficient of determination, *R*^2^, presents values that aew superior to 0.99. Despite of that, it is possible to observe that the best adjustments were achieved through the Sips and Langmuir models.

The values for adsorption parameters are summarized in Table 6.

Since the Sips and Langmuir models are the ones that better represent the experimental data, and the value of the ns parameter, obtained from the Sips model, is close to 1, the Langmuir model better represents the results that are obtained. In this sense, it is assumed that there is a homogeneous distribution of the active sites on the surface of the adsorbent (PVCDS).

**Figure 11:**
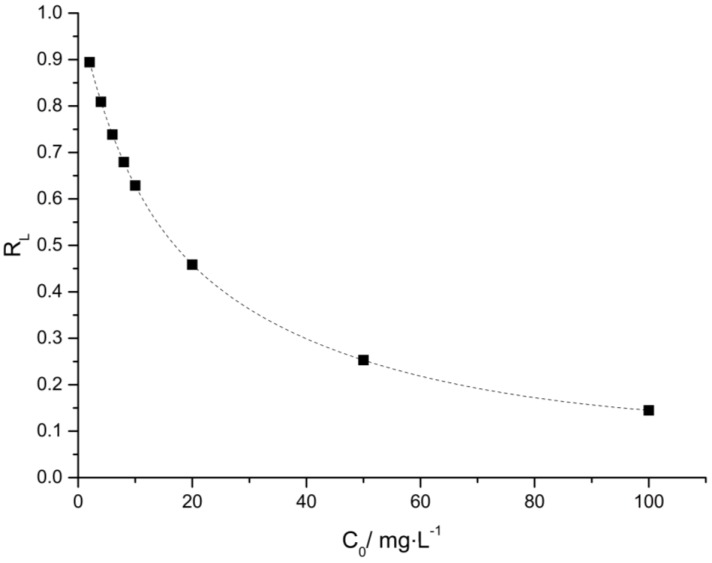
Separation factor as a function of the initial concentration of adsorbate.

Another important aspect in the evaluation of the adsorption mechanism considering the Langmuir model is the calculation of the constant *R_L_* Equation (2), separation factor found through the expression:

(2) $$R_{L}=\frac{1}{\left(1+K_{L}.C_{0}\right)}$$

in which *K_L_* is the equilibrium constant of Langmuir and *C*_0_ is the initial concentration of the dye (mg·L^−1^). Figure 11 shows calculated *R_L_* values at different initial dye concentrations. 

Dimensionless constant, *R_L_*, indicates the shape of the isotherms to be either unfavorable (*R_L_* > 1), linear (*R_L_* = 1), favorable (0 < *R_L_* < 1), or irreversible (*R_L_* = 0) [32]. In Figure 11, it was observed that *R_L_* values were determined between 0.9027 and 0.1445 for PVCDS dyes. This indicates that the adsorption was more favorable with the higher the initial dye concentrations than for the lower concentrations. However, the adsorption process is favorable throughout the range of concentrations evaluated.

As seen in Table 6, the maximum adsorption capacities for VL onto PVCDS for Langmuir and Sips models are 370.31 and 392.14 mg·g^−1^, respectively. There are few studies in the literature dealing with VL adsorption. Dezhampanah et al. (2013) [33], studied the removal of thionine dye (VL) from aqueous solutions using low-cost materials as adsorbent as rice husk. The results showed that VL was adsorbed efficiently in rice husk and the experimental data of equilibrium adsorption were better when adjusted through the Langmuir model. The rice husk presented good adsorption capacity of VL reaching the value of 8.67 mg·g^−1^. The values found in this work are higher than those observed by Dezhampanah and this result is related to the significant difference of chemical structure between the lignocellulosic material (rice husk) and the chemically modified PVC. Bulut and Aydin (2006) [34] investigated the methylene blue adsorption, a dye that has a similar chemical structure to VL, they verified that the maximum adsorption capacity depends strongly on the adsorbent, e.g., the adsorption in rice husk reached 40.58 mg·g^−1^ and in active coal, 435 mg·g^−1^.

The high adsorption capacity of the PVDCS is related to the chemical structure with the presence of a few sulfonic groups and the hydrophobic character of the polymeric chain, these two aspects, allied to the value of surface area can be the key to the favorable interaction and adsorption of the dye.

## 3. Materials and Methods 

### 3.1. Dehydrochlorination of the Polyvinyl Chloride Resin

The PVC chemical modification was carried out using an intermediate step for the chlorine removal. This stage, which is called dehydrochlorination, was adapted from the works presented in the literature [8,35,36]. With the intention of promoting the reaction of partial elimination of chlorine and producing a partially unsaturated intermediate, the poly(ethylene glycol) of molar mass 400 g·mol^−1^ (PEG-400) was used as a phase transfer catalyst, besides the solvent tetrahydrofuran (THF) and poly(vinyl chloride) resin (PVC). PVC and PEG were dissolved in THF in a w/w (%) ratio of 1:2:20, respectively. The system was maintained at 303 K in a thermostated bath under agitation until complete solubilization of PVC. Thereafter, under stirring, a solution of sodium hydroxide (KOH) (40%, w/w) at 1:1 w/w (KOH/PVC) was added slowly and held for 45 min under the same conditions of temperature and stirring. At the end of this period the material was washed with deionized water until the filtrate was fully cleared.

### 3.2. Sulfuric Acid Treatment of Polyvinyl Chloride Resin-Partially Dehydrochlorinated (PVCD)

The product of partial dehydrochlorination was obtained after the treatment with concentrated sulfuric acid. The temperature was maintained at 298 K under thermostatic bath and shaking for a period of 24 h. At the end of the previous step, the material was washed with deionized water in three cycles (24 h/cycle) to reach a pH range of 5–6 in the filtrate. After the washes with water, the material was immersed in anhydrous alcohol for the removal of possible residues adsorbed to the structure, and oven dried at 333 K for 24 h.

### 3.3. Characterization

All of the characterizations were performed with unmodified and chemically modified samples to verify the possible structural, functional, and physicochemical changes occurred on the material. The determination of carbon (C), hydrogen (H), and sulfur (S) was performed using an Elemental Analyzer 2400 CHN-Perkin Elmer equipment. 

The ion exchange capacity (ITC) was performed using the volumetric titration technique [37]. This technique aims to estimate the value of the available ionizable groups of the sample. Their results are expressed as millimols per gram (mmol·g^−1^) or milliequivalents per gram (mEq·g^−1^) of ionizable groups per mass of dry material.

To analyze the morphology of the samples, a scanning electron microscope Zeiss model EVO MA-10 was used. The samples were sprayed on a suitable metal support and was covered with gold by the sputtering technique.

The X-ray diffractograms of the samples were obtained on the XRD-6000 Shimadzu diffractometer, operating at a power of 40 kV with 30 mA of current, using CuK α radiation (1.5418 Å) with 2θ ranging from 5° to 37° and a scanning speed of 1 min^−1^ and resolution of 0.02°.

For the Fourier Transform Infrared Spectroscopy (FTIR) measurements, the samples were prepared as KBr pellets and analyzed using a Shimadzu IR Prestige-21 equipment. Thirty two scans with a resolution of 4 cm^−1^ were performed in the range between 400 and 4000 cm^−1^.

Thermogravimetric analyses were performed using a Shimadzu TGA-50H model equipment. About 7 mg of the sample was heated in aluminum crucibles to 600 °C at the heating rate of 10 °C·min^−1^, under a nitrogen atmosphere at 50 cm^3^·min^−1^.

The evaluation of the surface area (specific volume and mean pore diameter) was performed using an ASAP 2010 volumetric adsorption analyzer, manufactured by Micromeritrics, at 77 K (boiling point of N_2_). The samples were subjected to a vacuum at 298 K, reaching a residual pressure of 10 Pa^−4^. The Brunauer, Emmett, and Teller (BET) method was used to calculate the surface area and the pore size distribution.

### 3.4. Solutions and Reagents

In the present work, a commercial micronized poly(vinyl chloride) resin was used with an average molar mass of about 93.4 × 10^3^ g·mol^−1^. The resin was supplied by Polyvin, a Brazilian company specialized in the production of PVC pipes and fittings.

For the chemical modifications of the PVC-micronized resin, the following reagents were used: anhydrous tetrahydrofuran-THF (Sigma-Aldich, São Paulo, Brazil) (99.99%), poly(ethylene glycol)-PEG (Vetec, Rio de Janeiro, Brazil) molar mass 400 g·mol^−1^, anhydrous sulfuric acid (Vetec) (95–99%), potassium hydroxide-KOH (Modern Chemistry, São Paulo, Brazil), pellets (98%), and anhydrous ethanol (Vetec) (96–98%).

**Figure 12:**
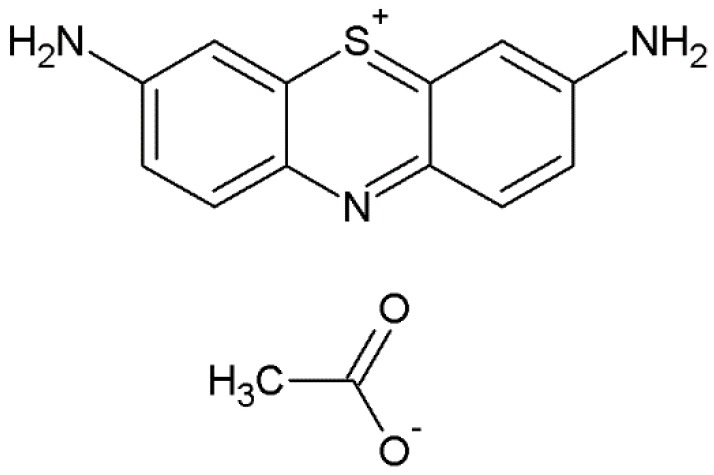
Molecular structure of thionine (Lauth’s Violet, VL).

For adsorption experiments, deionized water was used throughout all experiments to prepare the solutions. The dye acetate of 3,7-diamino-5-phenothiazine (dye used in microscopy), Lauth’s Violet (VL) or thiazine (C_14_H_13_N_3_O_2_S, CI-52,000, 287.34 g·mol^−1^, λ_max_ 597–601 nm, pH ~ 6.8 see Figure 12) was obtained from Lobal Chemie with a dye content of 85% and was used without further purification. The amino groups in the dye structure give the molecule basic ionization characteristics and the thioether is responsible to the positive charge. The stock solution was precisely prepared by dissolving the dye in distilled water to a concentration of 600 mg·dm^−3^. The working solutions were obtained from dilutions of stock solution of the dye.

### 3.5. Batch Adsorption Procedure 

Adsorption studies to evaluate the chemically modified poly(vinyl chloride) resin (PVCDS) for removal of VL from aqueous solutions were performed in triplicate using a batch adsorption process. Adsorbent amounts ranging from 5.0 to 50.0 mg were suspended in a series of 50 cm^3^ glass vials containing 25.0 cm^3^ of dye solution at concentrations ranging from 5.00 to 10.0 mg·dm^3^. These suspensions were stirred for suitable times in the range from 1 to 1440 min. The isotherms did not demonstrate the formation of a plateau, during the equilibrium studies, which was determined under optimized conditions of 30 min at 303 ± 1 K, with the initial pH of the dye solutions ranging from 2.0 to 10.0. Subsequently, to separate the adsorbents from the aqueous solution, the solutions were filtered under vacuum. For the system in question, it was observed high adsorption rates, and the maintenance of contact even for a short period of time caused significant changes in the amount of dye present in the system, therefore filtration was performed immediately after adsorption procedure was finished. Final concentrations of 1.0–10.0 cm^3^ aliquots of the filtrate were determined by visible spectrophotometry using a Femto Spectrophotometer, which was supplied with optical glass cells of 1.0 cm path length. Absorbance measurements were taken at the maximum wavelength of the VL dye (600 nm). The value of detection limit for VL used in the spectrophotometric method was determined according to the IUPAC at 0.005 mg·dm^−3^ [38]. The amount of dye absorption and the percentage of dye removal by the adsorbent were calculated applying Equations (3) and (4), respectively,

(3) $$q=\frac{C_{0}-C_{f}}{m\;}\cdot V$$

(4) $$\%R=100\cdot \frac{C_{0}-C_{f}}{C_{0}}\;$$

where *q* represents the maximum amount of dye adsorbed by mass of adsorbent (mg·g^−1^); *C*_0_ is the initial concentration of VL dye in contact with the adsorbent (mg·dm^−3^); *C_f_* is the concentration of the remaining dye after the adsorption procedure (mg·dm^−3^); *V* is the volume of the dye solution (dm^−3^); and, *m* is the mass (g) of adsorbent.

### 3.6. Kinetic and Equilibrium Models

The equations of the kinetic studies corresponding to the pseudo-first order, pseudo-second order and intraparticle diffusion models are given in Table 3 [30,39,40,41,42,43]. The equations corresponding to the isotherm models for Langmuir, Freundlich, Sips, and Redlich-Peterson were listed in Table 5 [44,45,46,47].

### 3.7. Statistical Evolution of Kinetic and Isothermal Parameters

The kinetic models were adjusted using the linearized functions, and the isothermal models were adjusted using non-linearized functions. The Microcal Origin 9.0 software was used for these adjustments.

## 4. Conclusions

The modification of PVC by chemical treatment with concentrated sulfuric acid of the partially dehydrochlorinated polymer was efficient in the production of a sulfonated polymer (PVCDS). The PVCDS showed 65% dehydrochlorination and a decrease of the C/S molar ratio of 120, in the original polymer, to 31 in the modified polymer, indicating the increase of the molar contribution of sulfur. The PVCDS had an ion exchange capacity of 1.030 mmol·g^−1^ due to the presence of sulfonic groups. 

Chemically modified PVC (PVCDS) is a good adsorbent for aqueous solutions of the cytological dye thionine (Lauth’s violet). The thionine adsorption on PVCDS reached equilibrium in 30 min. The adsorption kinetics of the VL in the PVCDS was better adjusted for a pseudo second order model. This result indicates that the VL adsorption onto PVCDS occurs by chemisorption. The equilibrium sorption data fitted the Langmuir and Sips isotherms models better than the Freundlich and Redlich-Peterson. PVCDS showed high adsorption capacities (370 mg·g^−1^). This aspect shows that PVCDS removes efficiently VL from aqueous solutions being a viable alternative in the removal of this emerging contaminant and in the employment of alternative adsorbents. 

**Figure 13:**
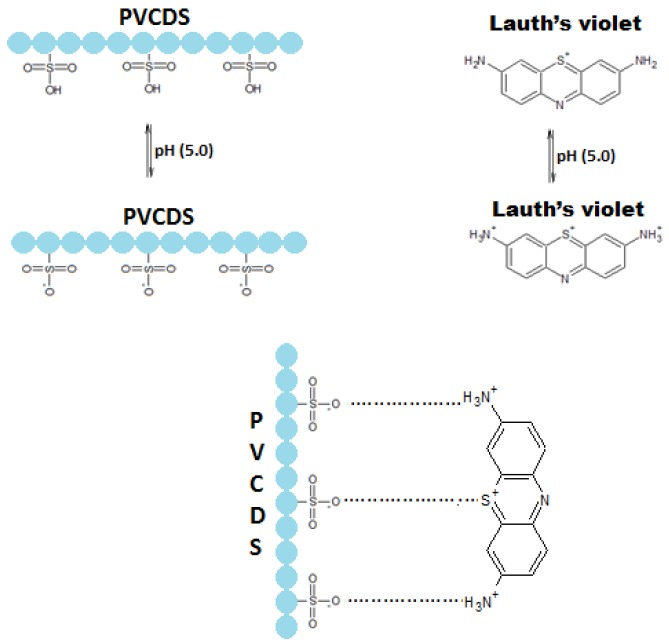
Proposition for the mechanism of interaction between the dye and the absorbent.

By the viewpoint of the solid/liquid phase exchanger process, the PVCDS, having sulfonic groups, interacts electrostatically with amine groups of the VL, attached to the aromatic rings (see Figure 13). This interactive process occurs at pH 5, when nitrogen basic atoms are protonated and the deprotonated sulfonic groups occurring as an interaction due to electrostatic forces. 

The results suggests that the dye uses more than one acid center from polymer structure, the interactive process, as could be expected, occurs through chemical interactions to neutralize opposite charges of the acid/base groups between the dye and the resin.

## Figures and Tables

**Table 1:** Mass percentage and the molar ratio of the elements, carbon, hydrogen, nitrogen, and sulfur.

Samples	Percentage (%) (m/m)					Molar Ratio				
	% C	% Other Elements	% H	% S	% N	C	Other Elements	H	S	N
**PVC**	34.75	59.68	4.68	0.76	0.13	2.89	1.68	4.64	0.024	0.0098
						C/S *	OE/S	H/S		
						120.4	70	193.3	1	
**PVCDS**	58.91	30.09	5.84	5.02	0.14	4.90	0.849	5.73	0.158	0.0100
						C/S *	OE/S	H/S		
						31.0	5.37	36.3	1	

* Molar ratio.

**Table 2:** Surface area, *σ*, specific pore volume, *V*, and mean pore diameter, *d*, for PVC and PVCDS samples.

Samples	Surface Area	Specifc Pores Volume	Avegere Pores Diameter
	*σ* (m^2^·g^−1^)	*V* (cm^3^·g^−1^)	*d* (nm)
**PVC**	0.27	0.0021	6.59
**PVCDS**	295.57	0.43	5.76

**Table 3:** Adsorption kinetics models.

Kinetic Models	Equations
Pseudo-first order	$\log\left(q_{e}-q_{t}\right)=\log q_{e1}-\frac{k_{1}}{2303}t$
Pseudo-second order	$\frac{t}{q_{t}}=\frac{1}{k_{2}qe^{2}}+\frac{1}{q_{e}}t$ Initial rate of adsorption $h=k_{2}qe^{2}$
Intraparticle diffusion	$q_{t}=k_{id}.t^{1/2}+C$

**Table 4:** Kinetic parameters for VL removal using PVCDS as adsorbent.

**Experimental Date**	**Value**
log *q_e_*_(exp)_	1.078 (±0.004)
*q_e_*_(exp)_	11.96 mg·g^−1^ (±0.12)
**Pseudo first-order**	
*q_e_*_1_	31.88 mg·g^−1^
*k*_1_	0.448 min^−1^
*R*^2^	0.9464
**Pseudo second-order**	
*q_e_*_2_	11.74 mg·g^−1^
*h*	15.49 mg·g^−1^·min^−1^
*k*_2_	0.1123 g·mg^−1^·min^−1^
*R*^2^	0.9994
**Intraparticle diffusion**	
*C*	9.95 mg·g^−1^
*k*_id_	0.0696 mg·g^−1^·min^1/2^
*R*^2^	0.6523

Conditions: temperature 303 K; pH 5.0 (±0.5); Adsorbent mass: 10 mg.

**Table 5:** Isotherm models.

Isotherm Models	Equations
Langmuir	$q_{e}=Q_{max}K_{L}C_{e}/\left(1+K_{L}C_{e}\right)$
Freundlich	$q_{e}=K_{F}C_{e}^{1/nF}$
Sips	$q_{e}=\;\frac{Q_{max}.Ks.Ce^{1/ns}}{1+Ks.Ce^{1/ns}}\;$
Redlich-Peterson	$q_{e}=\frac{K_{RP}.Ce}{1+a_{RP}.Ce^{g}},\;where\;0\leq g\leq 1$

**Table 6:** Parameters obtained from adsorption isotherm models for VL removal using PVCDS as adsorbent.

Model and Parameters			
**Langmuir**		**Freundlich**	
***Q_max_* (mg·g^−1^)**	370.9	*K*_F_ (mg·g^−1^(mg·dm^−3^)^−1/nf^)	25.48
***K_L_* (dm^3^·mg^−1^)**	0.0590	*n*_F_	0.694
***R*^2^**	0.9928	*R*^2^	0.9908
**Sips**		**Redlich-Peterson**	
***Q_max_* (mg·g^−1^)**	392.1	*a*_RP_ (mg·dm^−3^)^−g^	0.24557
***n*_s_**	1.0389	*K*_RP_ (dm^3^·g^−1^)	28.69
***Ks* ((mg·dm^−3^)^−1/nf^)**	0.0571	*G*	0.674
***R*^2^**	0.9941	*R*^2^	0.9924

Conditions: temperature 303 K; pH 5.0 (±0.5); Adsorbent mass: 10 mg.

## Notations

*a*_RP_	Redlich–Peterson constant (mg·dm^−3^)g^−1^
*C*	constant related with the thickness of the boundary layer (mg·g^−1^)
*C_f_*	dye concentration at the end of the adsorption (mg·dm^−3^)
*C_e_*	dye concentration at equilibrium (mg·dm^−3^)
*C*_0_	initial dye concentration put in contact with the adsorbent (mg·dm^−3^)
*g*	dimensionless exponent of Redlich–Peterson equation
*h*	the initial sorption rate (mg·g^−1^·h^−1^) of the pseudo-second order equation
*k*_1_	pseudo-first order rate constant (h^−1^)
*K*_F_	the Freundlich constant related to adsorption capacity ([mg·g^−1^(mg·dm^−3^)^−1/nf^])
*k*_id_	intraparticle diffusion rate constant (mg·g^−1^·h^−1/2^)
*K*_L_	Langmuir affinity constant (dm^3^·mg^−1^)
*K*_RP_	Redlich–Peterson constant (dm^3^·g^−1^)
*K*_S_	the Sips constant related to the affinity constant (mg·dm^−3^)^−1/ns^
*k*_2_	the pseudo-second order rate constant (g·mg^−1^·h^−1^)
*m*	mass of adsorbent (g)
*n*_F_	dimensionless exponent of the Freundlich equation
*n*_S_	dimensionless exponent of the Sips equation
*q*	amount of the dye adsorbed by the adsorbent (mg·g^−1^)
*q_e_*	amount of adsorbate adsorbed at the equilibrium (mg·g^−1^)
*q_t_*	amount of adsorbate adsorbed at time *t* (mg·g^−1^)
*q_e_*_1_	amount of adsorbate adsorbed at the equilibrium (mg·g^−1^) to the pseudo-first order model
*q_e_*_2_	amount of adsorbate adsorbed at the equilibrium (mg·g^−1^) to the pseudo-second order model
*Q*_max_	the maximum adsorption capacity of the adsorbent (mg·g^−1^)
*V*	volume of dye put in contact with the adsorbent (dm^3^)
*R_L_*	the separation factor of the Langmuir (mg·dm^−3^)
